# Study on Load Transfer Mechanism of Local Curved Prestressed Hollow-Core Slab Bridge

**DOI:** 10.3390/ma16134708

**Published:** 2023-06-29

**Authors:** Jihao Chen, Yuxin Wang, Qian Zhu

**Affiliations:** 1 School of Civil Engineering and Communication, North China University of Water Resources and Electric Power, Zhengzhou 450045, China; cjh@ncwu.edu.cn (J.C.);; 2 School of Civil Engineering and Architecture, Zhengzhou University of Aeronautics, Zhengzhou 450046, China

**Keywords:** hollow-core slab bridge, deep joint, local curve prestress, finite element analysis, force transmission mechanism

## Abstract

The assembled hollow-core slab bridge is the most widely used beam bridge in China. With the increasing traffic volume and traffic load in China, the joints of the hollow-core slab bridge are prone to damage. In this paper, a hollow-core slab bridge with locally curved prestressed tendons is proposed. Based on the static load test of a beam with joints taken from the cross section of a hollow-core slab bridge in practical engineering, a finite element nonlinear analysis is used to simulate the test, and the concrete and interface parameters under the correct analysis results are obtained. Finally, the parameters are applied to the three-beam and two-joint hollow-core slab bridge with a span of 10 m and a finite element analysis is carried out to explore the total failure process and performance improvement effect of the prestressed hollow-core slab bridge. The results show that the interface unit method can successfully simulate the new-to-old concrete interface where the joint is in contact with the precast beam segment. Compared with the static load test results, the analysis error of each finite element model is basically within 15%. Compared with the traditional hollow-core slab bridge, the cracking load, through-joint load, and ultimate load of the prestressed hollow-core slab bridge are increased by 50.0%, 91.7%, and 66.7%, respectively. Under the same load, the stress of the U-bar, the relative deflection of both sides of the joint, and the maximum width of the joint of the prestressed hollow-core slab bridge are lower than those of the traditional hollow-core slab bridge. When the ultimate load is reached, the longitudinal crack lengths of the traditional hollow-core slab bridge and the prestressed hollow-core slab bridge are 0.48 L and 0.4 L, respectively, and the damage degree of the prestressed hollow-core slab bridge is lower than that of the traditional hollow-core slab bridge.

## 1. Introduction

Assembled hollow-core slab bridges are widely used in China. Force is transmitted between hollow-core slabs through joints. However, most hollow-core slab bridges in China adopt joints without transverse ties, and the transverse connection between hollow-core slabs is weak [1,2]. With the increase in traffic load and traffic volume, many in-service hollow-core slab bridges have suffered structural damage [3,4]. Joints are the weak positions of hollow-core slab bridges. After the joints are damaged, the force transfer between hollow-core slabs will be affected, which will lead to the stress increase of a single hollow-core slab, and even only one hollow-core slab will bear the load [5,6]. Due to its simple design, convenient construction, and rapid installation and replacement, the application of unbonded prestressed tendons in concrete structures has attracted more and more attention [7,8,9]. Since there is no bond between prestressed tendons and concrete, the tedious process of grouting prestressed tendons is omitted. Compared with other reinforcement technologies, it has been proved that using unbonded prestressed tendons to strengthen concrete structures is one of the most economical technologies [10,11].

**Figure 1:**
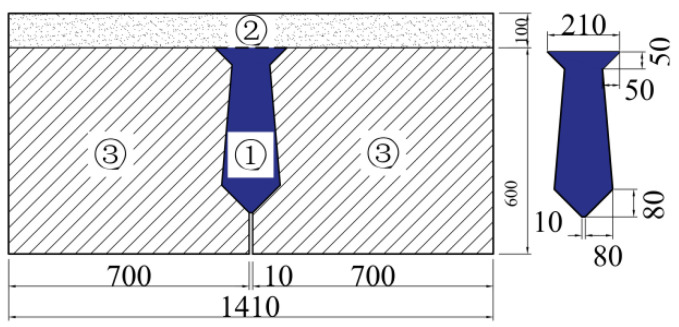
Specimen size. Note: ①-Joint; ②-Laminated layer; ③-Precast beam segment [12].

Joints with shear keys, joints without shear keys, and traditional joints (as shown in Figure 1 [12], which are composed of three trapezoids) are widely used in assembled hollow-core slab bridges. The performance of the shear key has an important influence on hollow-core slab bridges, and some scholars have studied the performance of the shear key. Kaneko et al. [13,14] developed the mechanical model of the single shear key joint, put forward the calculation formula of shear strength, and verified the formula through finite element analysis and experimental research. Lee et al. [15] studied joints with different numbers of shear keys. The results of finite element analysis show that the shear capacity of joints with double shear key is greater than that of joints with single shear key. In addition, increasing the height and decreasing the depth of the shear key can reduce the cracking load at the corner of the shear key. In addition, increasing the height and decreasing the depth of the shear key can reduce the cracking load at the corner of the shear key. Reginato et al. [16] put forward a numerical simulation method of shear keys and proved by finite element analysis that the interface strength increases with the increase of depth, length, and the number of the shear key. Kim et al. [17] conducted a shear test on the UHPC grouting joint, and found that the ultimate load of the joint with double shear keys increased by 1718 kN compared with the joint without shear keys, and the ultimate load of the joint increased by 44.5% when the height of the shear keys increased from 1.5 mm to 30 mm. Sangkhon et al. [18] found through a shear test that the ultimate load of joints with three shear keys is higher than that of corresponding joints with single shear keys. In addition, the average ultimate shear stress in descending order is the semicircular shear key, triangular shear key, and trapezoidal shear key. At present, the research on the performance of joints with or without shear keys has been sufficient, but these studies are not completely applicable to the most widely used traditional joints in China. The study of traditional joints has not received much attention, and there are few related studies. In China, with the use of assembled hollow-core slab bridges over time, many bridges have suffered damage, with the damage to joints being the most prominent. Therefore, the structural performance of traditional joints needs to be studied urgently.

Some scholars have studied the hollow-core slab bridge and its reinforcement methods. Yi et al. [19] tested hollow-core slab bridges with shallow joints, and the results showed that under eccentric load, the shallow joints were mainly subjected to shear stress, and the tensile stress at the bottom was very small. Di et al. [20] studied the shear performance of hollow-core slab bridges with deep joints. The results show that when a 15 cm thick post-cast roof concrete layer is used, the cracking load of 10 m, 16 m, and 20 m hollow-core slabs increases by 47%, 32%, and 8%, and the ultimate load increases by 41%, 22%, and 18%, respectively. Zou et al. [21] demonstrated through finite element analysis that the use of prestressed carbon fiber plates to strengthen hollow-core slab bridges can enhance the lateral connections between adjacent hollow-core slabs. Xiang [22], Liang [23], and Li [24] used prestressed tendons to strengthen hollow slabs. The finite element analysis and static load test results show that prestressed tendons can effectively uniformize the lateral distribution of loads. The integrity of the strengthened bridge is enhanced, the load transfer capacity is improved, and the bearing capacity of the bridge is improved. Jin et al. [25] strengthened a bridge with steel-reinforced concrete and carried out a static load test. The results show that the stiffness of the strengthened bridge is improved and the deflection is greatly reduced. Wang et al. [26] externally prestressed a hollow-core slab bridge with severely damaged joints. The test results show that with the increase of prestress, the deflection of each hollow-core slab decreases, and the mid-span deflection decreases by 38%. Ni et al. [27] carried out finite element analysis on a hollow-core slab bridge strengthened with steel beams, and the results showed that only adding a layer of steel beams at the bottom of the hollow-core slab bridge may lead to tensile stress at the upper end of the joint, while using double-layer steel beams and adding diaphragms can avoid this risk. Li et al. [28] studied the influence of the number of transverse prestressing tendons and the magnitude of prestress on the transverse connection performance of bridges. The results showed that the average values of the longitudinal distribution of the transverse prestress discount factor were 0.48 and 0.62 when single and three prestressing bars were used, respectively. In addition, the ultimate bearing capacity increases with the increase of transverse prestress. Li et al. [29] proposes the method of enlarging section combined with prestressed tendon (Enlarging Section-Prestress Method) to strengthen the bridge. The model test shows that the strengthening effect of the prestressed method with enlarging section-prestress method is obvious, and the ultimate bearing capacity of the beam is increased by 50%. Kim et al. [30] evaluated the effect of additional prestressing using fiber-reinforced polymers and strands applied to a demolished, deteriorated bridge. The test results show that the external prestressing method exhibited the highest stiffness (15 kN/mm) within the load range of 200–400 kN, followed by non-strengthening (8.5 kN/mm) and the near-surface mount method (5.45 kN/mm). The external prestressing method increased the stiffness by approximately two times. At present, many reinforcement methods have been proposed for hollow-core slab bridges, but the related literature lacks an analysis of the failure process of hollow-core slab bridges. To provide a better reference for the repair and reinforcement of bridges in practical projects, it is necessary to analyze the total failure process of hollow-core slab bridges.

To sum up, at present, the performance of shear keys in joints has been fully studied, but the related research can not be fully applied to the traditional joints used in China. In addition, there is little research on the failure process of hollow-core slab bridges in the retrieved related literature. In order to improve the durability of joints, this paper puts forward a hollow-core slab bridge with local curved prestressed tendons. Based on the nonlinear finite element analysis of static load test [12] of beams with joints, the damage plasticity and interface unit parameters of concrete are obtained. Then the nonlinear finite element model of the whole hollow-core slab bridge is established, and the failure process and performance improvement effect of the hollow-core slab bridge are explored, which provides more reference for the improved design, repair, and reinforcement of the hollow-core slab bridge.

## 2. Finite Element Analysis Method

**Figure 2:**
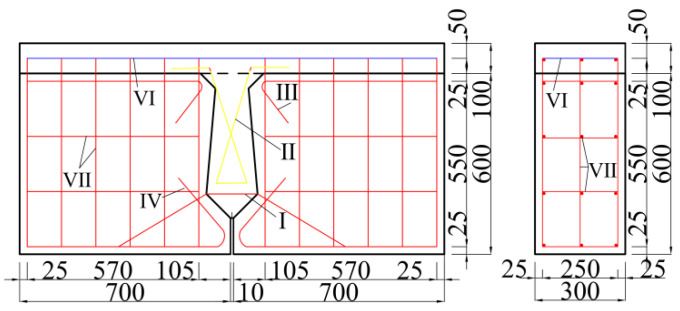
Reinforcement drawing. Ⅰ—U-bar; II—Cross rebar; III, IV—Construction rebar; VI—The rebar of the laminated layer; Ⅶ—The rebar of the precast beam segment [12].

The dimensions of the beam with the joint used in this paper are shown in Figure 1. The two sides of the beam are precast beam segments, and the middle is a post-pouring joint. This joint form is widely used in China and is called the traditional joint. A layer of concrete is poured on the beam to simulate the laminated layer in the hollow-core slab bridge. The reinforcement of the beam is shown in Figure 2 [12].

When the anchorage positions are the same, the length of the curved prestressed tendons is longer than that of the straight prestressed tendons. Therefore, the prestress loss caused by anchorage deformation is smaller. At the same time, Wells [31] tested the ultimate bearing capacity of segmental specimens strengthened with these two kinds of prestressed tendons, and the ultimate bearing capacity of straight tendon specimens is 60% of that of curved tendon specimens. Therefore, the curved prestressed tendon is designed as the local prestress technology in this study. In addition, the local curve prestress technology does not need to drill holes at the bottom of the hollow-core slab, and the pipes of the curved prestressed tendon and the grooves at the bottom of the hollow-core slab are reserved spaces before pouring. This method is a reinforcement technology when building a new bridge, which avoids the danger of drilling holes in the tendon. The specific method and test results have been described in reference [30].

### 2.1. Finite Element Model of Beam with Joint

#### 2.1.1. Concrete Setting

**Figure 3:**
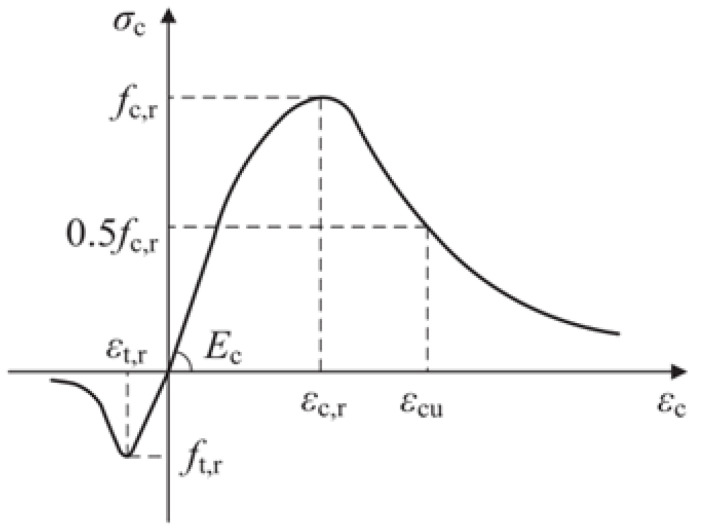
Concrete constitutive model.

**Figure 4:**
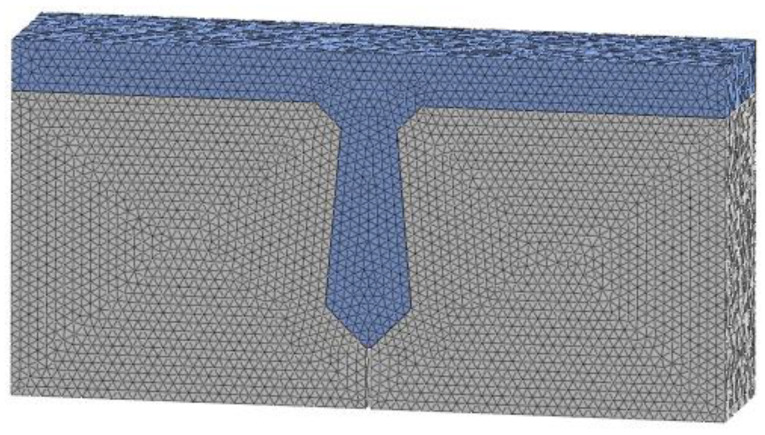
Schematic diagram of concrete grid.

Concrete with a design strength of 40 MPa is used for precast beam segments, and concrete with a design strength of 60 MPa is used for joints. For concrete, the damage plasticity model is used to simulate, and the model’s theoretical basis was put forward by Lubliner [32], Lee, and Fenves [33]. According to the China Code for Design of Concrete Structures [34], the constitutive relation of concrete is shown in Figure 3. The concrete is meshed by a 3D solid unit. The mesh size is an important index that affects the calculation results of the model. The larger mesh is easy to converge, but its error is large, while the finer mesh will lead to a long operation time and is not easy to converge. The grid size of concrete is 20 mm, and the grid division of concrete is shown in Figure 4.

#### 2.1.2. Rebar Setting

**Figure 5:**
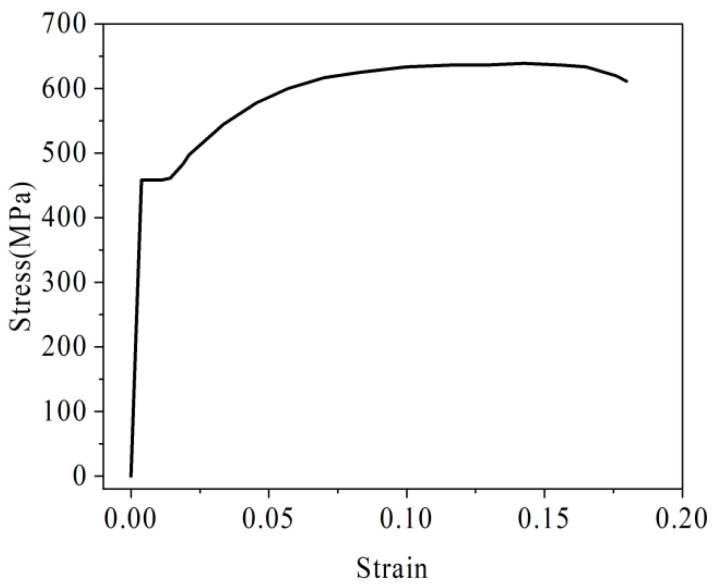
U-bar constitutive model.

**Figure 6:**
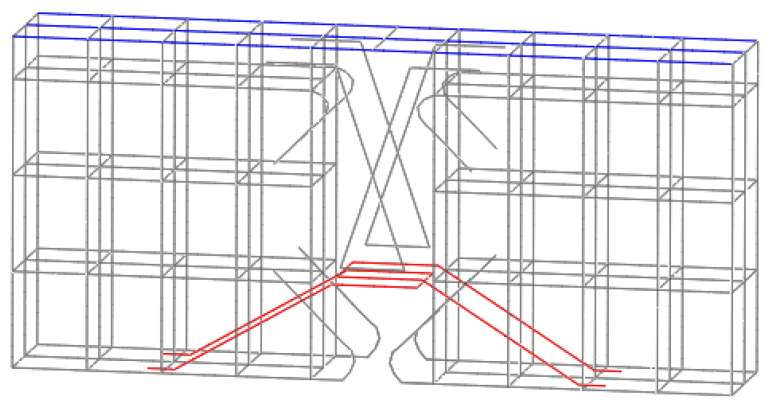
Schematic diagram steel skeleton grid.

Steel bars with a yield strength of 400 MPa are used in the model. Among them, the diameters of structural steel bars, U-bar, and steel bars in the laminated layer are 8 mm, 12 mm, and 10 mm, respectively. The rebar material is isotropic. In the nonlinear setting of steel bar materials, U-bar uses the stress-strain constitutive relation, and the motion criterion is used for analysis in the attribute set. According to the uniaxial tensile curve of steel bars in reference [35], the stress-strain constitutive model of steel bars is shown in Figure 5. The ideal plastic constitutive relation is used for other steel bars, and the yield stress is set to 400 MPa. All rebars are meshed by a 1D unit, and the unit attribute is an implanted truss element, which can automatically connect the nodes of steel bars and concrete. The rebar grid size is 20 mm, and the unit division is shown in Figure 6.

#### 2.1.3. Interface Setting of New-to-Old Concrete

**Figure 7:**
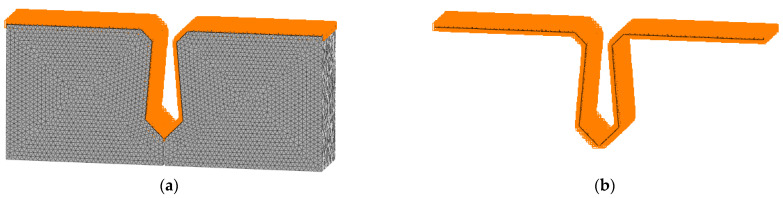
Interface unit settings, (**a**) Schematic diagram of new-to-old concrete interface, (**b**) Schematic diagram of interface unit.

For the surface between the precast beam segment and joint, the interface unit is added between the precast beam segment and joint to simulate. When the interface is added, the interface unit can automatically disconnect the node between the precast beam segment and the joint and add a layer of element surface without thickness between them. The constitutive model of the interface adopts the discrete crack model, to simulate the cracking of the new-to-old concrete interface. Its important structural parameters include normal interface stiffness, shear interface stiffness, tensile strength, and fracture energy, respectively. The schematic diagram of the interface unit is shown in Figure 7.

#### 2.1.4. Setting of Unbonded Prestressed Tendons

**Figure 8:**
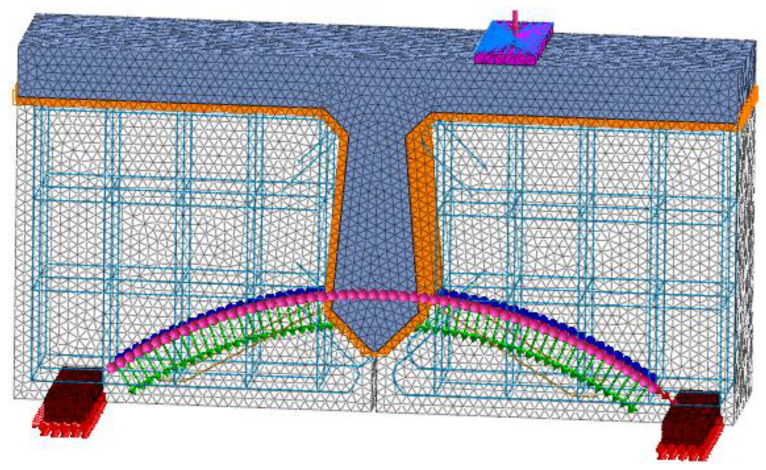
Model of prestressed tendon specimen.

This paper regards prestressed tendons as an elastic material, and its constitutive relation is linear elasticity. When setting the prestressing tendons, the prestressing input is 200 kN, which slightly exceeds the maximum allowable stress calculated according to 0.75f_tpk_. When dividing prestressed tendon elements, prestressed tendon nodes and concrete nodes will be automatically coupled. However, the prestress technology used in this paper is unbonded prestress, and the arrangement of prestressed tendons is curved. Referring to the setting method in references [36], this paper uses the coupling method of the local coordinate system to simulate the unbonded state of a curved prestressed tendon. First, the other nodes except the outermost grid nodes on the prestressed tendons are disconnected to make them free, and then each node is set as the origin in turn, and the X axis points to the next node, thus establishing the local coordinate system of the node. In the local coordinate system of each node, the displacement in the beam width direction is constrained, and the displacement in the X direction and the beam height direction is released, so that it can move along the prestressed tendons and the beam height direction. For the two outermost nodes of prestressed tendons, the coupling state with concrete is always maintained, to ensure that prestressed tendons and beams have the same movement. The schematic diagram of prestressed reinforcement is shown in Figure 8.

#### 2.1.5. Bearing and Constraint Setting

**Figure 9:**
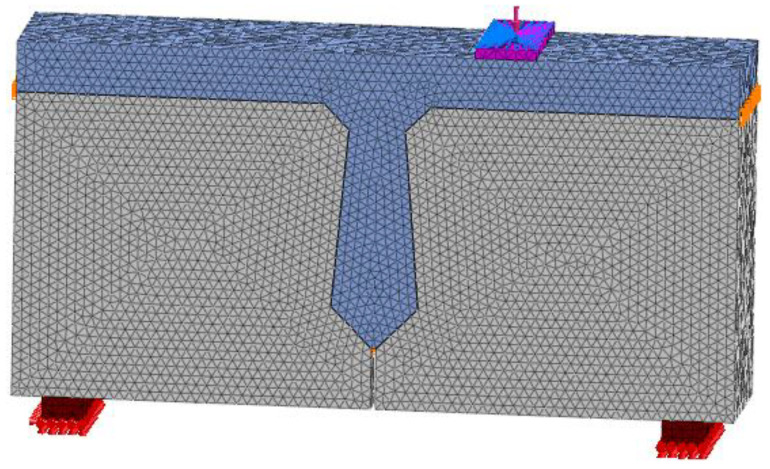
Schematic diagram of load, constraint, and pad.

One side of the beam adopts a hinged bearing, and the other side adopts a sliding bearing. The thickness of the bearing pad is 50 mm, and the distance from the edge of the beam section is 100 mm. A node is established on one side of the beam segment 250 mm away from the joint center, and the node is rigidly connected with other nodes of the pad on the same plane before applying load, thus eliminating the influence of stress concentration. The thickness of the pad under load is 20 mm. The schematic diagram of load, constraint, and pad is shown in Figure 9.

**Figure 10:**
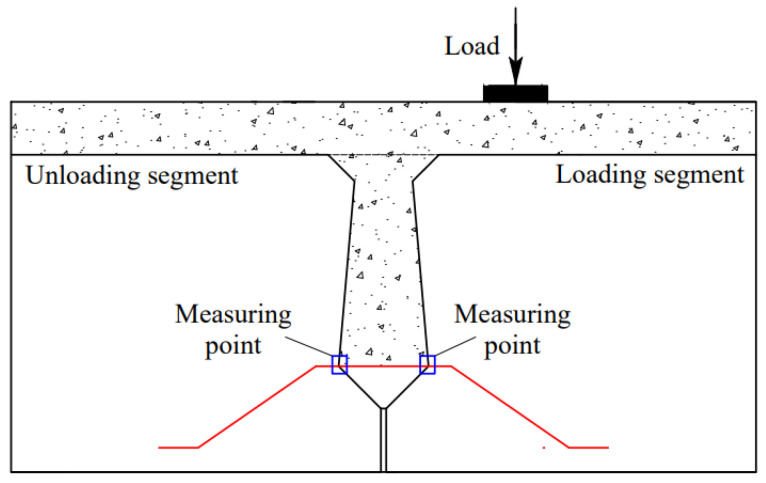
U-bar arrangement.

**Figure 11:**
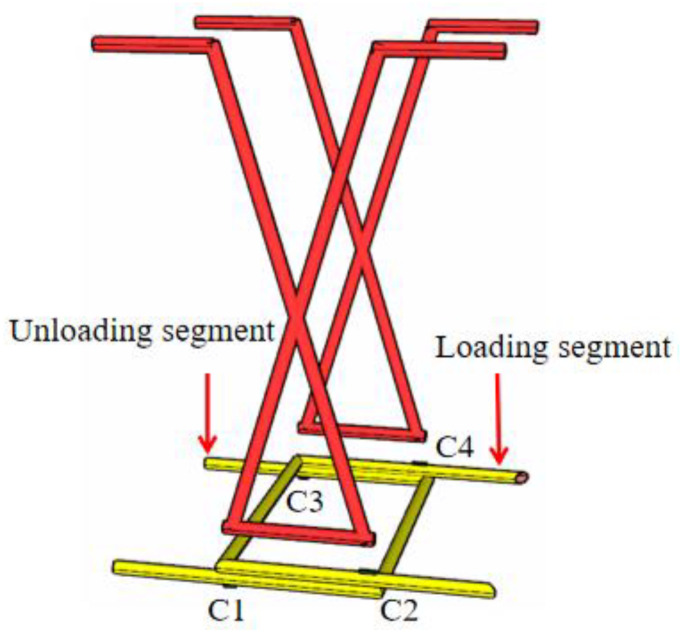
Layout of steel bar measuring points.

The loading side is called the loading segment, and the non-loading side is called the unloading segment. The U-bar is arranged 200 mm away from the bottom of the beam, as shown in Figure 10. Steel bar measuring points are arranged at the intersection of U-bars and joint concrete, as shown in Figure 11. The rebar stress in the loading segment is obtained by averaging C2 and C4, and the rebar stress in the unloading segment is obtained by averaging C1 and C3.

### 2.2. Hollow-Core Slab Bridge Model and Loading

**Figure 12:**
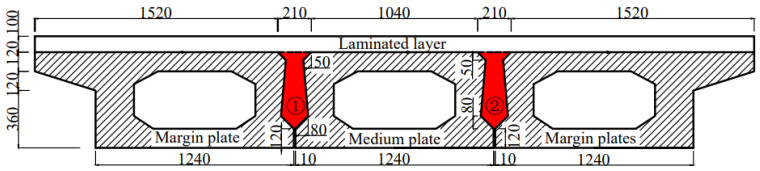
Cross-sectional dimensions of a hollow-core slab bridge. Note: ①-Joint I; ②-Joint II.

A hollow-core slab bridge with a span of 10 m is selected for modeling. The hollow-core slab bridge consists of three hollow-core slabs and two joints, and its specific dimensions are shown in Figure 12.

**Figure 13:**
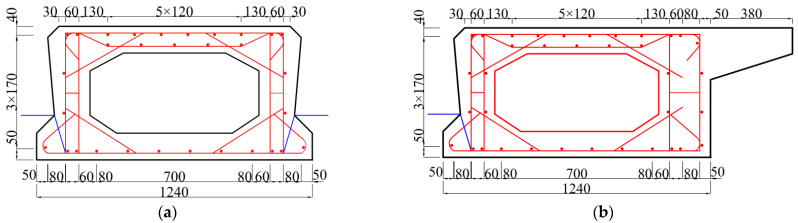
Reinforcement of hollow-core slab, (**a**) Reinforcement of medium plate, (**b**) Side plate reinforcement.

In the model of a hollow-core slab bridge, the concrete with design strength of 40 MPa and 60 MPa is still used for the hollow-core slab and joint, respectively. Since the hollow-core slab is a hollow-core structure, it is slightly different from the reinforcement in the beam with a joint. The reinforcement of the hollow-core slab is shown in Figure 13. 

**Figure 14:**
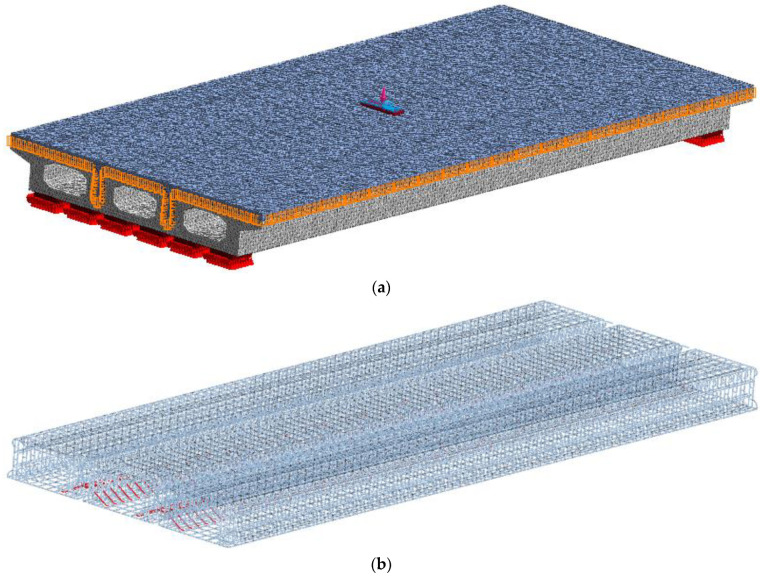
Hollow-core slab bridge grid, (**a**) Concrete grid. (**b**) Steel skeleton grid.

When the finite element model of a hollow-core slab bridge is established, the modeling method and constitutive relation are the same as those of the beam with a joint. According to Chen Jihao’s invention patent, curved unbonded prestressed tendons are set at the joints. According to the most unfavorable position of load, the load is applied in the span of the hollow-core slab bridge. According to the contact area between the standard vehicle and the road surface, the load pad is 0.2 m long and 0.6 m wide. One side of each hollow-core slab is sited with hinge bearings, and the other side is sited with sliding bearings. The length, width, and height of the bearing are 400 mm, 400 mm, and 100 mm, respectively. Due to the large volume of the hollow-core slab bridge, the grid size of the concrete is set to 50 mm and the grid size of the steel bar is set to 20 mm. The grid of the hollow-core slab bridge is shown in Figure 14.

According to the static load test of the beam with joint, two finite element models of the beam with joint are established in this paper to verify their correctness and analyze them. At the same time, the finite element models of two hollow-core slab bridges with three beams and two joints are established to analyze their failure process and performance improvement effect. The specific numbers of finite element models are shown in Table 1.

## 3. Model Verification and Result Analysis

### 3.1. Model Verification

Through repeated verification, the interface parameters of beams B-C and B-P are determined, respectively. The normal stiffness of the beam B-C interface is 12,000 MPa, the tangential stiffness of the interface is 120 MPa, and the tensile strength is 1 MPa. The normal stiffness of the beam B-P interface is 20,000 MPa, the tangential stiffness of the interface is 200 MPa, and the tensile strength is 0.6 MPa. Under the set parameters, the cracking load, through-joint load, and ultimate load of each beam are shown in Table 2.

**Figure 15:**
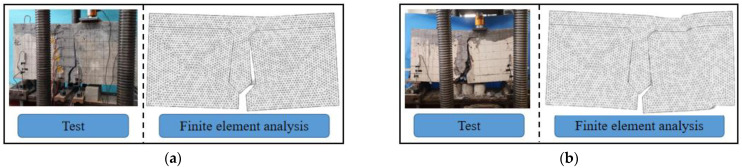
Failure pattern of beam, (**a**) Beam B-C, (**b**) Beam B-P.

It can be seen from Table 2 that the cracking load, through-joint load, and ultimate load of each beam are in good agreement with the test values under the set parameters. The cracking load of beam B-C is only 10 kN different from the test value, the through-joint load is only 20 kN different from the test value, and the ultimate load is consistent with the test value. The cracking load of beam B-P differs from the test value by 30 kN, and it is speculated that the possible reason is that the crack at the bottom of the interface was not observed during the test, resulting in a higher cracking load. The through-joint load of beam B-P is only 20 kN different from the test value, and the ultimate load is only 10 kN different from the test value. The failure mode of each beam is consistent with the reference [30], as shown in Figure 15.

**Figure 16:**
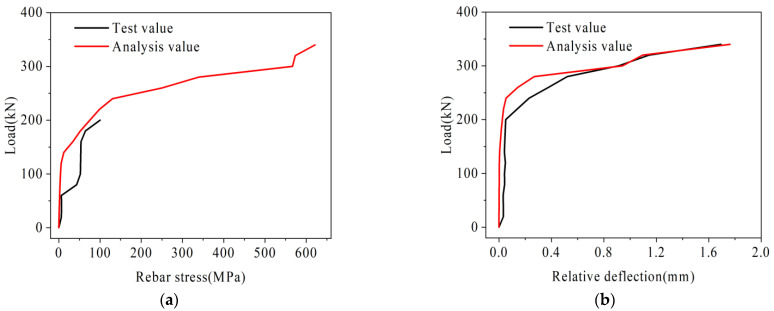
Beam B-C analysis result diagram, (**a**) Load-reinforcement stress diagram, (**b**) Load-relative deflection diagram.

**Figure 17:**
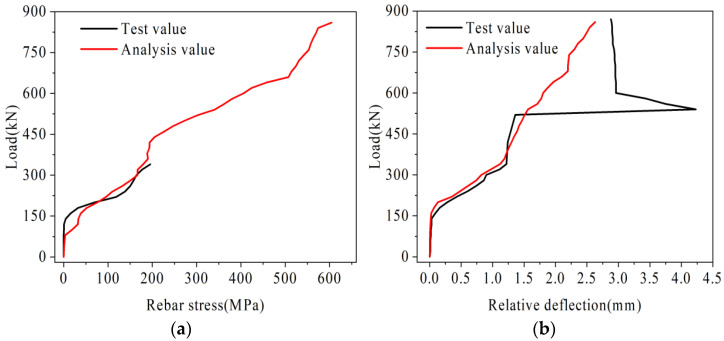
Beam B-P analysis result diagram, (**a**) Load-reinforcement stress diagram, (**b**) Load-relative deflection diagram.

The rebar stress in the loading segment and the relative deflection of joints obtained from the analysis of each beam are shown in Figure 16 and Figure 17.

As shown in Figure 16, the variation law of rebar stress and relative deflection in beam B-C is basically consistent with the test. Under the load of 340 kN, the rebar stress is 621 MPa, which basically reaches its ultimate bearing capacity. In addition, because the deformation of beam B-C is too large at this time, the finite element calculation is no longer convergent. Under the ultimate load, the relative deflection in the test results is 1.69 mm, and the relative deflection in the finite element analysis results is 1.76 mm, with an error of only 4.1%.

As can be seen from Figure 17, the variation law of rebar stress of beam B-P is basically consistent with the test results. Under the load of 340 kN, the rebar stress in the test value is 195.4 MPa, and that in the analysis value is 179.2 MPa, with an error of only 8.2%. Under the load of 860 kN, the rebar stress is 604.58 MPa, which is basically close to its ultimate bearing capacity. Ignore the relative deflection mutation caused by the sudden instability of the beam due to the fracture of the U-bar in the unloading segment [30]. Under the ultimate load, the relative deflection in the test results is 2.88 mm, and that in the finite element analysis results is 2.63 mm, with an error of only 9.5%.

### 3.2. Interface Stress Analysis

**Figure 18:**
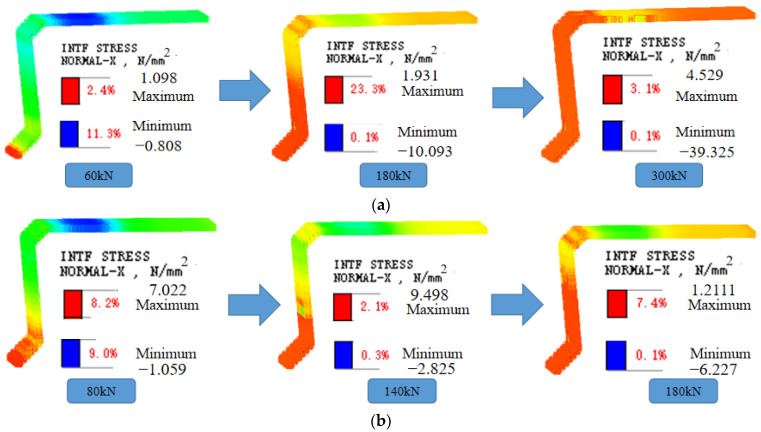
Stress nephogram of interface element, (**a**) Beam B-C, (**b**) Beam B-P.

The development of stresses at the interface on the loading side of beams B-C and B-P is shown in Figure 18. The beams B-C and B-P show similar force processes. At initial loading, the lower part of the interface unit is subjected to tension and the upper part to compression. As the load continues to increase, the tensile force of the interface unit gradually increases, while the height of the tensile zone gradually rises. With the growth of interfacial unit tension, the interfacial unit cracks, and cracks gradually extend upward. Eventually, the height of the tensile zone reaches the bottom of the laminated layer and the interface unit is completely cracked.

Beams B-C and B-P reach the through-joint condition at 300 kN and 200 kN, respectively. Under 200 kN load, the height of the tensile zone at the beam B-C interface is 452.2 mm, while beam B-P has formed a through-joint. It can be seen that the presence of prestressing makes the bottom tensile stress first cancel with the pre-pressure in the beam when the beam is subjected to the load, which makes the cracking load of beam B-P increase by 42.9%. However, the prestressing tendons did not inhibit the deterioration rate of the interface after the cracking of the interface unit, which is consistent with the results of the cracking load and through-joint load of beam B-C and beam B-P measured at the time of the test.

### 3.3. Stress Analysis of Joint and Laminated Layer

**Figure 19:**
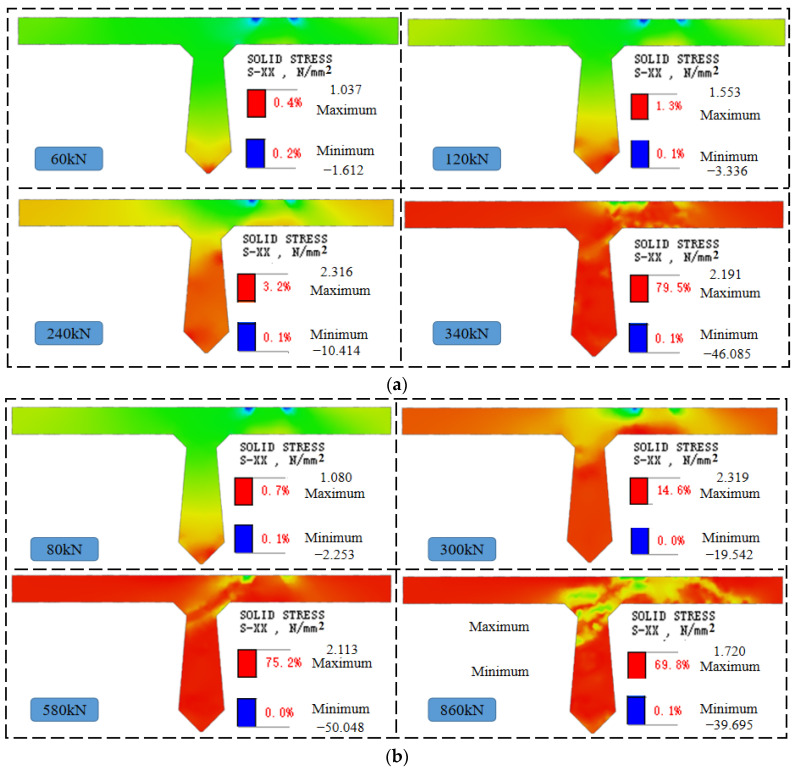
Stress nephogram of joint and laminated layer, (**a**) Beam B-C, (**b**) Beam B-P.

The stress variation clouds of joints and laminated layers of each beam are shown in Figure 19.

As shown in Figure 19, the transverse stress development process of the joint is similar for both beams. When beams B-C and B-P are initially loaded, the joints show a stress state with the lower part under tension and the upper part under compression. As the load increases, the transverse tension of the joint increases, and the height of the tensioned area rises. The stresses in the joint concrete and the height of the tension zone of beam B-C are higher than those of beam B-P under the same load. For example, under 200 kN load, the stress in the joint concrete of beam B-C at a height of 330 mm is 0.99 MPa, while the stress in the joint concrete of beam B-P at the same height is 0.57 MPa, which is only 57.6% of that of beam B-C. In addition, under 300 kN load, the height of the joint concrete tension zone of beam B-C has reached 600 mm, while the height of the joint concrete tension zone of beam B-P is 491.0 mm, which is 18.2% lower than that of beam B-C. It can be seen that the prestressing tendons in beam B-P share the load, which reduces the transverse tensile stress of joint concrete and the rising speed of the tension zone height.

## 4. Hollow-Core Slab Bridge Results Analysis

### 4.1. Failure Process Analysis of Traditional Hollow-Core Slab Bridge

Under the load, the failure process of joint I of a hollow-core slab bridge is similar to that of joint II. Therefore, the failure process of joint I of each hollow-core slab bridge is analyzed, respectively.

**Figure 20:**
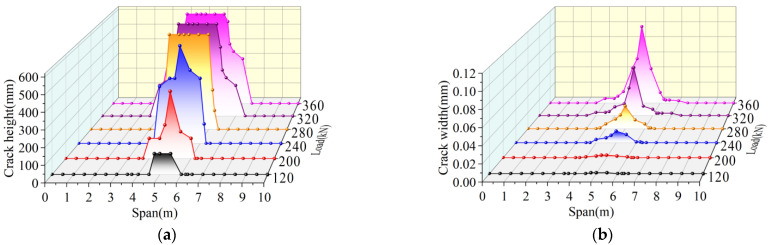
Development trend of cracks in traditional hollow-core slab bridge, (**a**) Crack height development, (**b**) Crack width development.

The crack development trend of joint I in a traditional hollow-core slab bridge is shown in Figure 20. When the load reaches 120 kN, cracks occur near the middle span, and the longitudinal length of the cracks is 0.6 m. The initial crack width is 0.001 mm and the initial crack height is 120 mm. When the load reaches 240 kN, the longitudinal crack span is from 4 m to 6 m. At this time, the mid-span crack extends upward to the bottom of the laminated layer, which is four times higher than the initial crack height. The width of the mid-span crack in the laminated layer increases to 0.018 mm, which is seventeen times higher than the initial crack width. When the load reaches 320 kN, the longitudinal crack span is from 2.2 m to 7 m. At this time, the cracks from 4 m to 6 m form a through-joint, and the width of the crack in mid-span increased to 0.069 mm, which was 2.83 times larger than that at 240 kN. When the load reaches 360 kN, the width of the crack in mid-span increases to 0.094 mm, which is 36.2% higher than that of 320 kN. At this time, the crack widths at 3 m, 4 m, 6 m, and 7 m are increased by 20%, 26.6%, 50.0%, and 14.2%, respectively, compared with those at 320 kN.

To sum up, the traditional hollow-core slab bridge cracks first in the mid-span under the load. As the load increases, the tensile stress at the bottom of the hollow-core slab bridge increases, and the crack width gradually increases, among which the crack width in the mid-span grows the fastest. With the increase in the distance from the mid-span, the increased speed of crack width at different spans gradually decreases. In addition, as the load increases, the height of the tensile zone rises, the tensile stress is transferred to both sides, and the cracks extend upward and to both sides, with the fastest growth rate of the crack height in the mid-span. With the increase in the distance from the mid-span, the upward extension speed of cracks at different spans slows.

**Figure 21:**
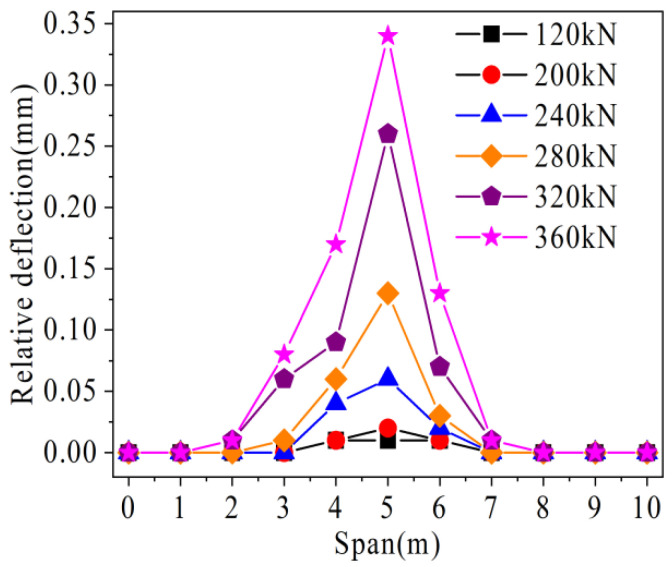
Load-relative deflection of traditional hollow-core slab bridge.

The relative deflection change of the traditional hollow-core slab bridge is shown in Figure 21. With the increase of load, the relative deflection at different spans increases gradually. This is because with the increase of load, the joint is gradually damaged, the force transmission performance is gradually reduced, and the vertical shear deformation on both sides of the joint is inconsistent. In addition, with the increase of load, the growth rate of relative deflection in the mid-span is the fastest. The relative deflection development rate decreases rapidly as the distance from the mid-span increases. For example, when the load is 120 kN, the relative deflection at 5 m and 4 m is 0.01 mm and 0.01 mm, respectively. When the load increases to 200 kN, 240 kN, 280 kN, 320 kN, and 360 kN, respectively, the relative deflection at 5 m increases one time, five times, twelve times, twenty-five times, and thirty-three times than that at 120 kN, respectively. The relative deflection at 4m is zero times, three times, five times, eight times, and sixteen times greater than that at 120 kN, respectively. In addition, the relative deflection of 3 m at 280 kN is only 0.01 mm, and as the load increases to 360 kN, the relative deflection increases only seven times. It can be seen that the vertical relative shear deformation of joints in the mid-span is the largest under the same load. With the increase in the distance from the mid-span, the relative vertical shear deformation of joints gradually decreases, which corresponds to the phenomenon of crack development.

**Figure 22:**
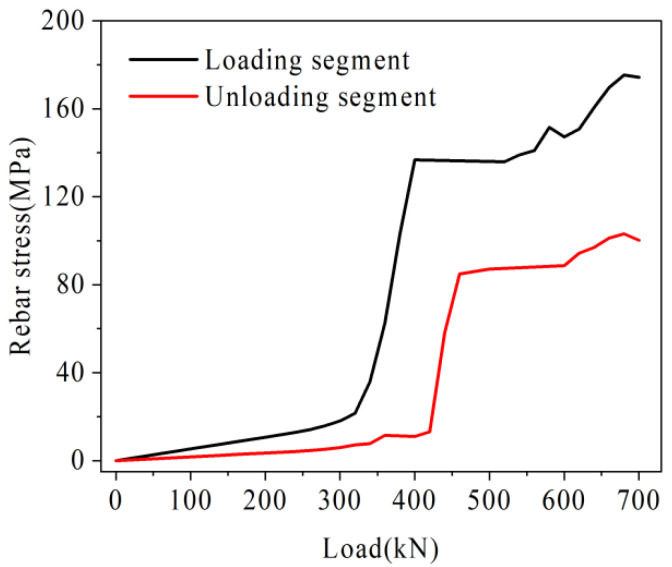
Load-rebar stress of traditional hollow-core slab bridge.

The change of U-bar stress at the mid-span of a traditional hollow-core slab bridge is shown in Figure 22. With the increase of load, the U-bar stress increases gradually. This is because with the increase of load, the interface cracks and gradually loses its force transfer function, while the U-bar shares the load transfer, which leads to the increase of its deformation and stress. In addition, the U-bar stresses in the loading segment are higher than those in the unloading segment under the same load. For example, under a load of 350 kN, the U-bar stress in the loading segment is 222.8 MPa and the U-bar stress in the unloading segment is 163.2 MPa, which is only 73.2% of the loading segment. This is because the loading segment is closer to the point of load action and the bottom tensile stress is higher than the unloading section. As shown in Figure 21, the U-bar stresses show a ‘slow-fast-slow’ increase process as the load increases. The “fast” growth section is approximated from the point where the crack height reaches the height of the U-bar to the point where the crack height reaches the bottom of the laminated layer. This shows that the damage to the U-bar in the mid-span is not a simple linear accumulation. The U-bar is damaged slowly until the crack height reaches the U-bar height. After the crack extends upward to the height of the U-bar, the U-bar exerts a tensile effect and the stress increases rapidly. After the crack height reaches the bottom of the laminated layer, the damage to the U-bar in the mid-span is slowed as the crack height at the other spans reaches the U-bar height and the U-bar at the other spans exerts its tensile effect.

### 4.2. Failure Process Analysis of Prestressed Hollow-Core Slab Bridge

**Figure 23:**
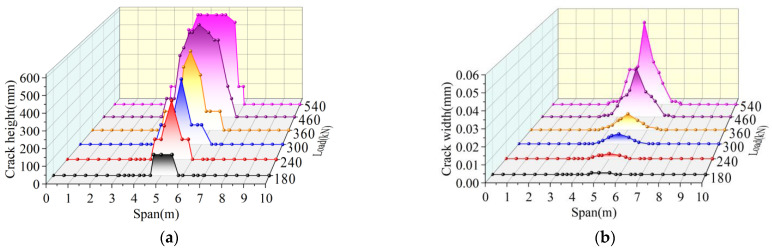
Development trend of cracks in prestressed hollow-core slab bridge, (**a**) Crack height development, (**b**) Crack width development.

The development trend of cracks in joint I of the prestressed hollow-core slab bridge is shown in Figure 23. When the load reaches 180 kN, cracks occur near the middle span, and the longitudinal length of the cracks is 0.85 m. The initial crack width is 0.001 mm and the initial crack height is 120 mm. When the load reaches 240 kN, the longitudinal cracks extend to 4.2 m and 5.8 m on both sides, respectively. At this time, the height of cracks in the mid-span increases by 191.6%, and the width of cracks increases by two times. When the load reaches 460 kN, the longitudinal crack span ranges from 3.3 m to 6.7 m. At this time, the mid-span crack extends upward to the bottom of the laminated layer, and the crack width increases to 0.031 mm, which is 14.5 times higher than that of 240 kN. When the load reaches 540 kN, the longitudinal cracks extend to 3.05 m and 6.95 m on both sides, respectively, and the cracks from 4.5 m to 6 m all form through-joints. At this time, the width of the mid-span crack increased to 0.054 mm, which was 74.2% higher than that of 460 kN. Under the ultimate load, the longitudinal crack span of the prestressed hollow-core slab bridge is only from 3.05 m to 6.95 m, and the longitudinal crack length is shorter than that of the traditional hollow-core slab bridge. It can be seen that prestress slows the damage speed of hollow-core slab bridges.

**Figure 24:**
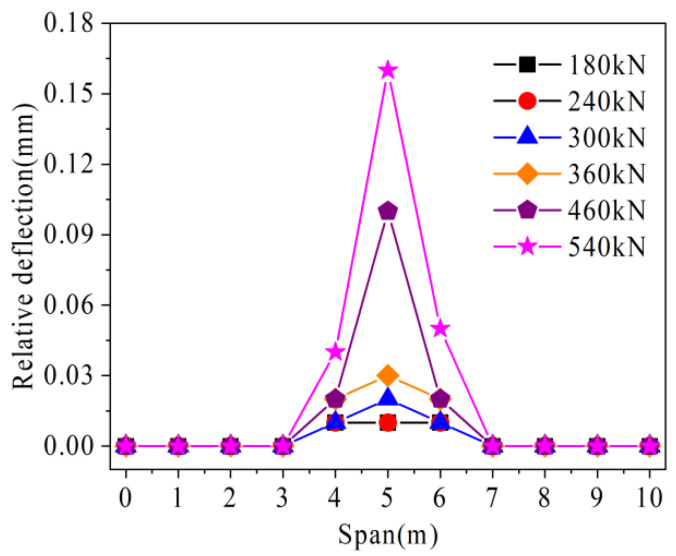
Load-relative deflection of prestressed hollow-core slab bridge.

The relative deflection change of the prestressed hollow-core slab bridge is shown in Figure 24. With the increase of load, the relative deflection in the mid-span increases rapidly, and the farther away from the mid-span, the slower the relative deflection increases. For example, when the load increases from 180 kN to 540 kN, the relative deflection in the mid-span increases from 0.01 mm to 0.16 mm. However, under the load of 540 kN, the relative deflections at 4 m and 6 m are 0.04 mm and 0.05 mm, respectively, which are only 25.0% and 31.3% of the mid-span relative deflections, respectively. For the uncracked parts, such as 0 m, 1 m, 2 m, 8 m, 9 m, and 10 m, because there is no crack at the interface, the force transmission performance of the joint is good, and there is no deflection difference between the connected hollow-core slabs.

**Figure 25:**
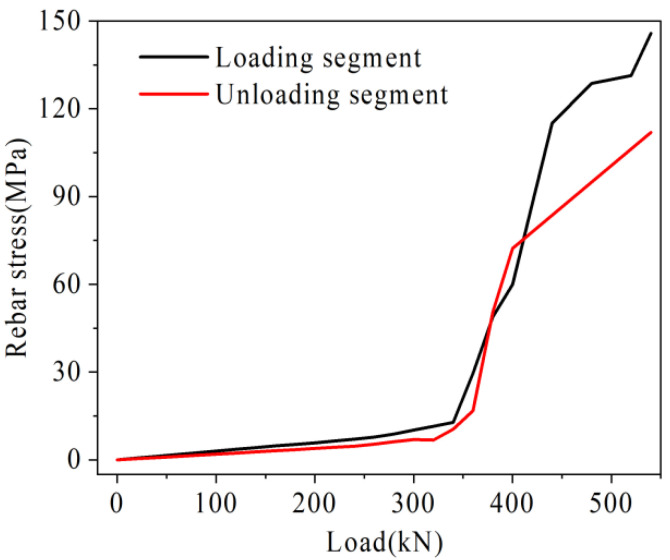
Load-rebar stress of prestressed hollow-core slab bridge.

The stress change of the U-bar in the prestressed hollow-core slab bridge is shown in Figure 25. With the increase of load, the stress of the U-bar increases gradually. In addition, the stress of the U-bar in the loading segment is generally higher than that in the unloading segment. For example, under the load of 540 kN, the U-bar stress in the loading segment is 145.8 MPa, and that in the unloading segment is 111.9 MPa, which is 76.7% of that in the loading segment. This is because the tensile stress at the bottom of the loading segment is higher than that of the unloading segment. With the continuous increase of load, the U-bar stress bars began to accelerate after experiencing slow growth, and its load inflection point was about 300–400 kN. It is speculated that the possible reason is that, on the one hand, after the crack height reaches the height of the U-bar, the U-bar bears tensile force, which leads to a rapid increase in stress. On the other hand, when the load is small, the local pre-compression stress in the beam counteracts part of the tensile stress. With the increase of load, the tensile stress at the bottom increases, and the contribution of pre-compression stress decreases, which leads to the rapid increase of U-bar stress.

### 4.3. Comparative Analysis of Results

**Figure 26:**
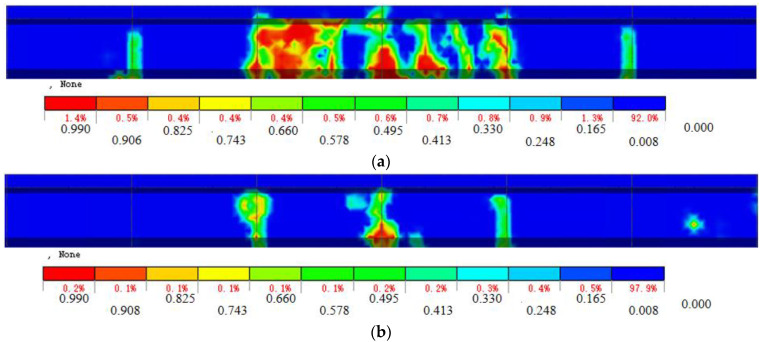
Nephogram of tensile damage of joint concrete, (**a**) Hollow-core slab bridge M-C, (**b**) Hollow-core slab bridge M-P.

The cracking load, through-joint load, and ultimate load of each hollow-core slab bridge are shown in Table 3. Since in each hollow-core slab bridge, the concrete damage of joint I is similar to that of joint II, and the damage is between 0.2 L and 0.8 L. Therefore, taking joint I one as an example, the tension damage clouds of joint concrete between 0.2–0.8 L for each hollow-core slab bridge under ultimate load are shown in Figure 26.

As can be seen from Table 3, compared with the hollow-core slab bridge M-C, the cracking load, through-joint load, and ultimate load of the hollow-core slab bridge M-P are increased by 50.0%, 91.7%, and 66.7%, respectively. At the same time, as can be seen from Figure 26a,b, under the ultimate load, the damaged span of joint concrete of hollow-core slab bridge M-C is from 2.2–7.0 m, while that of hollow-core slab bridge M-P is from 3.05–6.95 m, and the damaged span of joint concrete is reduced by 0.9 m. This is because in the hollow-core slab bridge M-P, the prestressed tendons provide local pre-compression stress for the joint concrete, and when subjected to load, the tensile stress generated at the bottom of the beam first offsets the pre-compression stress. At the same time, after the joint is cracked, the prestressed tendon can cooperate with the U-bar to transfer the load, which reduces the damage rate of the joint and enhances the lateral force transfer performance.

#### 4.3.1. Analysis of Rebar Stress

**Figure 27:**
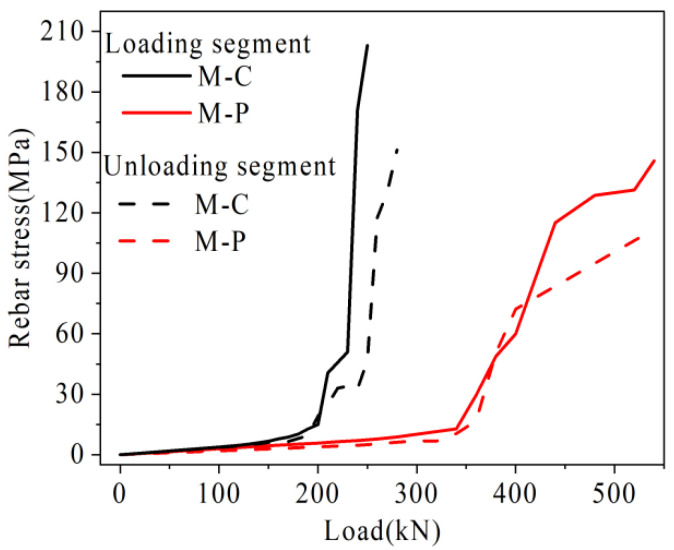
Load-rebar stress comparison diagram.

**Figure 28:**
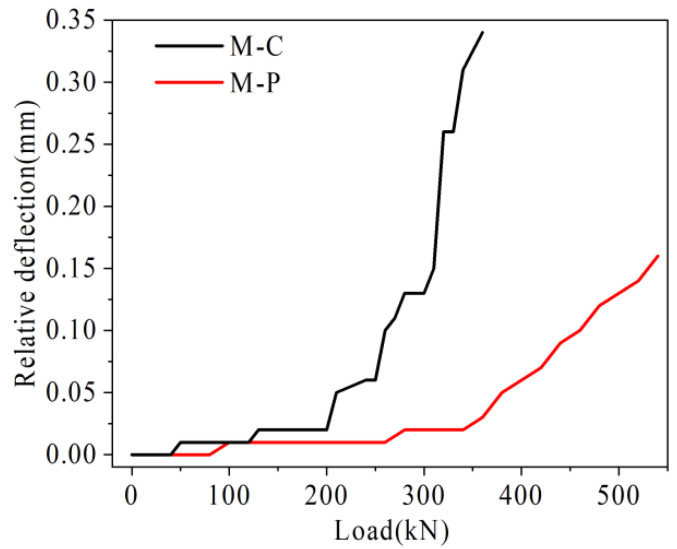
Load-relative deflection comparison diagram.

**Figure 29:**
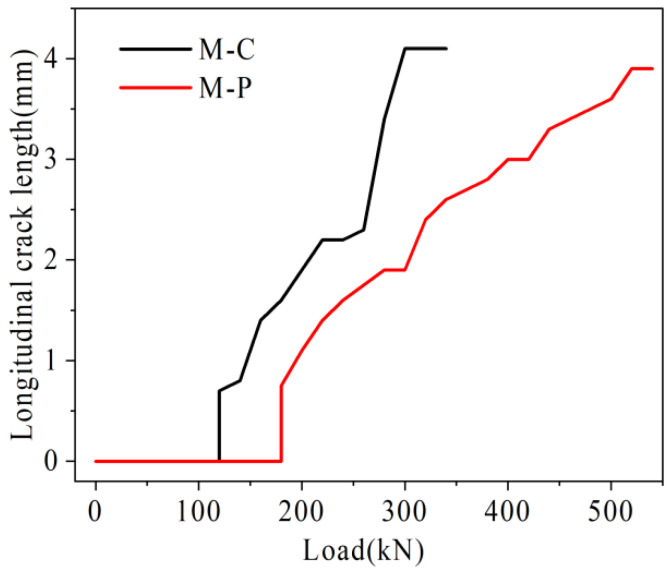
Load-longitudinal crack length.

Under the load, the damage rate of joints in the mid-span is the fastest. Therefore, the U-bar stress in mid-span, relative deflection of the joint, and the maximum width in mid-span in each hollow-core slab bridge joint I are plotted in Figure 27, Figure 28 and Figure 29.

As shown in Figure 27, under the same load, the rebar stress of hollow-core slab bridge M-C is higher than that of hollow-core slab bridge M-P. For example, under the load of 360 kN, the rebar stresses in the loading and unloading segment of hollow-core slab bridge M-C are 222.8 MPa and 163.2 MPa, respectively, while the rebar stresses of hollow-core slab bridge M-P are 29.6 MPa and 16.8 MPa, which are only 13.3% and 10.3% of those of hollow-core slab bridge M-C, respectively. It can be seen that in the prestressed hollow-core slab bridge after the joint is cracked, the prestressed tendon and the U-bar jointly transfer the load, which reduces the stress level of the U-bar under the same load.

#### 4.3.2. Relative Deflection Analysis

As shown in Figure 28, with the increase of load, the relative deflection growth rate of hollow-core slab bridge M-C is higher than that of hollow-core slab bridge M-P. For example, under the load of 360 kN, the relative deflection of hollow-core slab bridge M-C is 0.34 mm, while that of hollow-ore slab bridge M-P is 0.03 mm, which is only 5.8% of that of hollow-core slab bridge M-C. It can be seen that in the prestressed hollow-core slab bridge, under the load, the local pre-compression stress can offset some tensile stress, which reduces the damage of joints under the same load. At the same time, the prestressed tendons restrain the growth of vertical relative shear deformation at the joint by sharing the transfer load.

#### 4.3.3. Analysis of Longitudinal Crack Length and Maximum Crack Width

**Figure 30:**
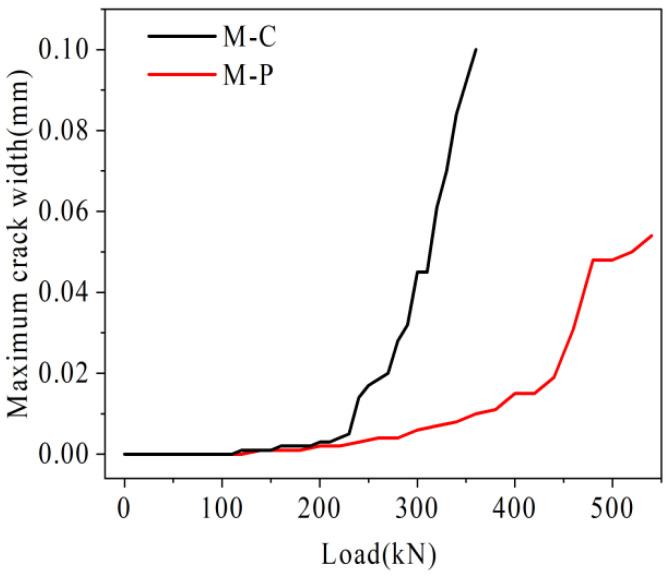
Load-Maximum crack width comparison diagram.

As shown in Figure 29, under the same load, the longitudinal crack length of hollow-core slab bridge M-P is lower than M-C. For example, under the load of 280 kN, the longitudinal crack length of hollow-core slab bridge M-C has reached 4.1 m, while the longitudinal crack length of hollow-core slab bridge M-P is 1.9 m, which is only 46.3% of that of hollow-core slab bridge M-C. As shown in Figure 30, with the increase of load, the maximum crack width of hollow-core slab bridge M-C increases faster than that of hollow-core slab bridge M-P. For example, under the load of 360 kN, the maximum width of cracks in hollow-core slab bridge M-C is 0.10 mm, and that in hollow-core slab bridge M-P is 0.01 mm, which is only 10.0% of that in hollow-core slab bridge M-C. On the one hand, the existence of prestressed tendons increases the total stiffness of tensile rebar and inhibits the growth of crack width. On the other hand, prestressed tendons share the transfer load, which reduces the stress level of U-bars. The deformation of U-bars decreases, which slows the growth of cracks.

### 4.4. Force Transmission Mechanism

Through the numerical simulation of hollow-core slab bridges, the total failure process of each hollow-core slab bridge can be divided into three stages:

(1) Uncracked stage: At this stage, no cracks are generated in the hollow-core slab bridge and the force performance of the joints is good. In this stage, with the load increases, the U-bar stress value, the relative deflection values on both sides of the joints, and the maximum crack width values grow slowly, and the load is mainly transferred by the interface unit.
(2) Working stage with cracks: At this stage, cracks are generated in the hollow-core slab bridge. With the increase of load, longitudinal cracks gradually extend to both sides of the hollow-core slab bridge, and the height of cracks gradually rises. With the increase of load, the stress value of the U-bar, the relative deflection value on both sides of the joint, and the maximum crack width increase faster than that in the uncracked stage. In this stage, in the traditional hollow-core slab bridge, the load is mainly transmitted by U-bars and interface units. In the prestressed hollow-core slab bridge, the load is mainly transmitted by U-bars, prestressed tendons, and interface units.
(3) Failure stage: At this stage, the cracks in the hollow-core slab bridge have reached the bottom of the laminated layer. With the increase of load, the stress value of the U-bar, the relative deflection value on both sides of the joint, and the maximum crack width value increase rapidly. In this stage, the interface unit in the hollow-core slab bridge exits to transfer load. In the traditional hollow-core slab bridge, the load is only transmitted by U-bars. In the prestressed hollow-core slab bridge, the U-bars and prestressed tendons jointly transfer the load.

## 5. Conclusions

In this paper, the local curved prestressed hollow-core slab bridge is proposed, and the finite element analysis is carried out according to the static load test of the beam with a joint. On this basis, the whole bridge model is numerically simulated, and the following conclusions are drawn:

(1) The interface normal stiffness of the traditional and prestressed hinge joint finite element models is 12,000 MPa and 20,000 MPa, respectively, and the interface tensile strength is 1 MPa and 0.6 MPa, respectively. The error between the finite element analysis results and the static load test results is basically within 15%.
(2) The total failure process of the prestressed hollow-core slab bridge is cracking near the mid-span under 33.3% ultimate load. Under 44.4% ultimate load, the longitudinal crack length reaches 1.6 m. Under 85.2% ultimate load, the longitudinal crack length reaches 3.4 m. Under 100% ultimate load, the longitudinal crack length reaches 3.9 m. With the increase of load, the longitudinal cracks extend to both sides and the width of cracks increases.
(3) Compared with the traditional hollow-core slab bridge, the cracking load, through-joint load, and ultimate load of the prestressed hollow-core slab bridge are increased by 50.0%, 91.7%, and 66.7%, respectively. The durability and bearing capacity of the prestressed hollow-core slab bridge have been improved.
(4) Under the same load, the U-bar stress, the relative deflection on both sides of joint, and the maximum width of joint of the prestressed hollow-core slab bridge are smaller than those of the traditional hollow-core slab bridge. Under the ultimate load, the longitudinal crack span of the traditional hollow-core slab bridge is 0.22–0.7 L, and that of the prestressed hollow-core slab bridge is 0.3–0.7 L. Prestressed tendons can share the transfer load and restrain the deterioration of a hollow-core slab bridge.

## Figures and Tables

**Table 1:** Finite Element Model and Test Beam Number.

Number	Moulded Dimensions	Enhancement Mode	Model Use
B-C	Scale model	Unreinforced	Finite element model verification
B-P	Scale model	local prestress	Finite element model verification
M-C	Hollow-core slab bridge model	Unreinforced	Finite element result analysis
M-P	Hollow-core slab bridge model	local prestress	Finite element result analysis

**Table 2:** Joint cracking through joint and ultimate load value.

Number	Cracking Load (kN)		Through-Joint Load (kN)		Ultimate Load (kN)	
	Test Value	Analysis Value	Test Value	Analysis Value	Test Value	Analysis Value
B-C	60	70	280	300	340	340
B-P	130	100	180	200	870	860

**Table 3:** Comparison table of hollow-core slab bridge results.

Number	Cracking Load (kN)	Through-Joint Load (kN)	Ultimate Load (kN)
M-C	120	240	360
M-P	180	460	540

## Data Availability

Data requirements can be directed to corresponding author.
